# Applying Concepts of Curriculum Design and Cultural Adaptation: Collaborating on a Dual-Degree Occupational Therapy Program in Mainland China

**DOI:** 10.1155/2024/1088666

**Published:** 2024-03-18

**Authors:** Julie McLaughlin Gray, Ling Yu (Elena) Meng, Adley Chan, Cameron Chang, Yijun Liu, Liguo Qian, Hui Wang, Ninghua Wang, Yanyan Yang, Mouwang Zhou, Grace T. Baranek

**Affiliations:** ^1^Chan Division of Occupational Science and Occupational Therapy, University of Southern California, Los Angeles, California 90089, USA; ^2^Gallagher Pediatric Therapy, 233 Orangefair Mall, Fullerton, California 92832, USA; ^3^Peking University First Hospital, Beijing 100034, China; ^4^Peking University Third Hospital, Beijing 100191, China

## Abstract

Occupational therapy is a profession with origins rooted in Western values. As culture plays an important role in shaping theory and practice, the curriculum design of academic programs that train future rehabilitation professionals should reflect the local context. As part of an international partnership, a dual-degree graduate program in occupational therapy was established between a Chinese and an American university. A team composed of members from both institutions collaborated on culturally adapting an entry-level master's program in occupational therapy for China, based on a U.S. program, which welcomed its first cohort in September 2019. This article details the timeline and process of program design and adaptation from conception, through implementation to evaluation and revision, with the aim of offering a framework for curriculum adaptation of other academic programs in the U.S. and internationally. The adapted curriculum includes the program mission, vision, and philosophy; the curriculum model with program outcomes and threads; the program scope and sequence; materials and resources; and course-specific objectives, learning activities, and assessments. The authors also share lessons learned through this experience of international collaboration as well as next steps for program evaluation and sustainability. The detailed overview of this international collaboration offers suggestions for individuals and institutions seeking to develop global partnerships and adapt curricula across cultural contexts.

## 1. Introduction: Mission and Vision of the USC Chan *China Initiative*

In 2014, the Mrs. T.H. Chan Division of Occupational Science and Occupational Therapy at the University of Southern California (USC Chan) launched the Chan *China Initiative* to address the immediate and consequential need to improve standards for the educational preparation of, and clinical care provided by, occupational therapists in Mainland China. In recent years, the Chinese government has consistently increased annual funding for rehabilitation services, including occupational therapy, in efforts to improve the health and well-being of persons with disabilities in China. The *China Initiative* was created to support and complement this goal, with a vision for a future in which occupational therapy in Mainland China is internationally recognized as both an academic and professional field of the highest quality.

To realize this vision, USC Chan explored collaborations with four top universities in China and ultimately entered into a formal partnership with Peking University Health Science Center (PKUHSC) to develop a graduate program in occupational therapy. These collaborative efforts to build a solid and substantive bridge between the USC Chan Division and a top-tier university program in China have guided the establishment of a rigorous master's degree program in occupational therapy which will become fully self-sufficient within 10 years. This degree program trains Chinese graduates to provide the highest caliber of occupational therapy services, with the ultimate aim to positively impact the quality of life and health for the Chinese population, including 85 million people with disabilities. This article provides a detailed account of how both institutions worked collaboratively to apply concepts of curriculum design and cultural adaptation in the development and launch of a dual-degree program between USC Chan and PKUHSC.

## 2. Background

### 2.1. Partnership between USC and PKUHSC

Early efforts of the Chan *China Initiative* led to a formalized partnership with PKUHSC, and a Memorandum of Understanding (MOU) with three key components was signed by the two institutions in October of 2016. At the core was an agreement to support PKUHSC to develop the first graduate-level degree program offered by a Mainland Chinese university in either rehabilitation therapy, with an occupational therapy (OT) emphasis, or occupational therapy outright.

As the first component of the MOU, USC agreed to share the Chan master's program curriculum and to provide faculty mentorship and training in curriculum design. Both parties concurred that the master's program created at PKUHSC should match USC's quality standards and would therefore be viewed as equivalent to the master's degree program in occupational therapy at USC, and standards of the Accreditation Council for Occupational Therapy Education (ACOTE) were used as guideposts. Given the short timeline until launch of the PKUHSC program in September of 2019, the partners decided to retain a similar curriculum scope and sequence in the PKUHSC master's program (as the Chan master's program) and make the cultural adaptations to content within each course. Consequently, the USC course sequence was mapped onto the PKUHSC academic calendar as much as possible for the program's first iteration with the expectation that ongoing curriculum revisions would occur and that PKUHSC would be responsible for continued cultural adaptation of the curriculum and content to the Chinese context.

The second component addressed the shared goal of developing the profession of occupational therapy in China through the design of a dual-degree program in which graduates of the PKUHSC master's program would be eligible for admission to the postprofessional Occupational Therapy Doctorate (OTD) degree program at USC. Once the master's program was launched, the MOU stipulated that at least five students each year, for the first three years of the PKUHSC master's program, would come to USC for the OTD program with full support and would return to China to further contribute to the development of OT at PKUHSC and the OT profession overall.

Finally, USC agreed to provide training to the inaugural instructors and future faculty who would deliver the curriculum at its launch in 2019 and also support the establishment of occupational science at PKUHSC. While the curriculum and “training plan,” or curriculum design, were being developed for proposal to the PKUHSC Graduate School, two students were selected to enroll in the USC master's and OTD programs in occupational therapy and to return to China as the first instructors of the PKUHSC master's program. Their doctoral residencies were specifically curated to prepare them for their instructional roles and therefore combined additional exposure to OT clinical practice in the U.S. with further training in pedagogy, including curriculum design, course development and cultural adaptation, and in-class teaching-related experiences. Additionally, two students from PKUHSC were also subsequently and one-by-one enrolled in the USC PhD program in occupational science (OS). The first graduated in 2022 with a focus on using ultrasound imaging to demonstrate the effects of everyday activities involving repetitive hand use on the median nerve, and the second is currently enrolled and focusing her doctoral work on the Lifestyle Redesign® evidence-based OT intervention approach to health promotion and chronic disease management developed at USC Chan.

### 2.2. Backward Curriculum Design

While the PKUHSC master's program was modeled after the scope and sequence of the USC master's program, principles of *backward curriculum design* also informed the collaborative curriculum development work, along with standards of the Accreditation Council for Occupational Therapy Education (ACOTE). Although most experts agree that curriculum design is dynamic, interactive, open-ended, and cyclical [1], a *backward design* process is widely regarded as the gold standard for developing curriculum in higher education. *Backward curriculum design* begins with identifying curriculum outcomes, or what faculty would like the students to “look like” upon graduation, followed by supporting the design with relevant evidence and learning theories, identifying learning objectives, and ending with learning activities and assessments [2]. Faculty from both institutions, along with the instructors-in-training at USC, held numerous discussions on the relevance of the USC program outcomes and ultimately modified the outcomes to better suit the PKUHSC context. The curriculum threads, a component of curriculum design required by ACOTE [3] and defined as “areas of study and development that follow a path through the curriculum and represent the unique qualities of the program, as demonstrated by the program's graduates” (p. 48), were also adapted in advance of course development. Through a collaborative process described in detail below, the identified outcomes for the PKUHSC program and curriculum threads were revised to better align with the PKUHSC educational philosophy and Chinese cultural values. Together with course-level learning objectives, these program outcomes and threads provided a framework for the design and cultural adaptation of course content and specific learning activities.

### 2.3. Needs and Best Practices for Cultural Adaptation

To further support the process of curriculum development and adaptation, the team reviewed literature on the challenges and needs associated with cross-border curriculum collaborations, as well as best practices to develop and sustain contextually relevant educational partnerships. Commonly identified areas for consideration included language differences, contextual relevance of the curriculum and learning materials, and teaching methods. Students may initially face language barriers when the language of instruction is different from their prior education [4], as well as in the fieldwork context if there is a mismatch between the language in which they studied and the local language spoken by clients [5]. The curriculum itself always needs to be adapted, given the change in the sociopolitical context of the educational experience, ranging from healthcare systems to legislation and political demands [4, 6]. If not enough attention is given to these adaptations, the curriculum may not be culturally or socially relevant and graduates may not be prepared to meet the local needs of the population [7, 8]. Additionally, learning materials, such as textbooks, are often developed from the perspective of the dominant culture and may not be reflective of the local context [5]. Finally, differences between teaching styles and assessment format should also be addressed, such as shifting from teacher-centered to learner-centered approaches [4, 6].

In order to address some of these areas, the following suggestions were given as best practices to develop and sustain contextually relevant educational partnerships: faculty development, adaptation of curriculum and learning materials, and access to resources and support systems. It is important to involve both instructors and clinical educators in continuous and intensive faculty development in such topics as the principles of the curriculum and a student-centered didactic model [5]. The curriculum should be assessed for elements not relevant to learning outcomes of the local students, which should be omitted or redesigned to better fit the context and intended learning objectives. Similarly, learning materials should be adapted such that original names, places, and circumstances are more recognizable and applicable to local students [5], or to examples that focus on universal approaches that can be applied in any context [8]. Finally, additional support and resources can be helpful for students who are adapting to the new teaching style or language of instruction [5]. The above considerations were influential in the development of the USC-PKUHSC dual-degree program, as faculty worked to address the language, cultural, and contextual differences between the American and Chinese educational programs.

## 3. Process of Developing the USC-PKUHSC Dual-Degree Program in Occupational Therapy

**Figure 1:**
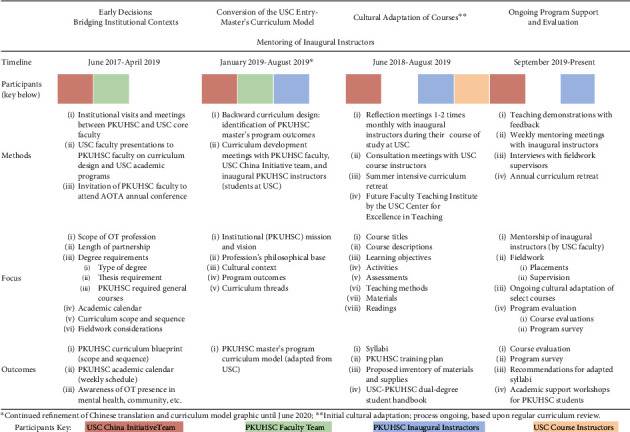
Cultural adaptation process map.

Figure 1 provides a visual representation of the major activities involved in the collaborative process between USC and PKUHSC to address all components of the MOU and ultimately ensure the sustainability of a high-quality and culturally relevant academic program. The timeline is divided into four main segments, with detailed methods, foci, and outcomes associated with each. Although each segment is presented in a sequential timeline with corresponding months and years, the actual work reflected an iterative process with overlapping segments and time frames. Key participants at each stage varied, as depicted by the color-coding, and included the Chan *China Initiative* team, including doctoral residents at USC; the PKUHSC Faculty Team; the PKUHSC Inaugural Instructors; and other USC Chan course instructors. Each of these segments is described in detail throughout the remaining sections of this paper.

### 3.1. Early Decisions: Bridging Institutional Contexts

This first phase of the collaboration began approximately two years prior to the official launch of the dual-degree program and involved relationship-building and mutual teaching and learning by faculty from both PKUHSC and USC. The leadership met for several in-person meetings to review the academic programs offered at USC Chan, principles of curriculum development, the types of degrees conferred by PKUHSC, and the curriculum requirements for a master's level program. The Chan faculty also prioritized the sharing of an expanded vision for the profession of occupational therapy to include mental health and community-based services, given occupational therapy's links in China to rehabilitation therapy and almost exclusive focus on hospital-based adult physical rehabilitation. In support of this goal, Chan invited and hosted faculty from PKUHSC to attend the American Occupational Therapy Association (AOTA) annual conference, which commemorated the 100th anniversary of the profession in 2017. Following the AOTA conference, the USC team also added Chinese subtitles to the “Celebrating AOTA's Centennial: A Historical Look at 100 Years of Occupational Therapy” and shared the translated version in presentations at several clinical service departments and academic conferences in China, including students and educators at the Rehabilitation Medicine Department of the Peking University (PKU) First Hospital and Third Hospital, as well as at the Third China Rehabilitation Medicine Forum, all in June 2017. All of these efforts were aimed at engaging the PKUHSC collaborators more broadly and experientially with the occupational therapy professional community, at further extending their awareness of the scope of present-day occupational therapy practice, and at making the historical AOTA video accessible to Chinese-speaking practitioners.

**Figure 2:**
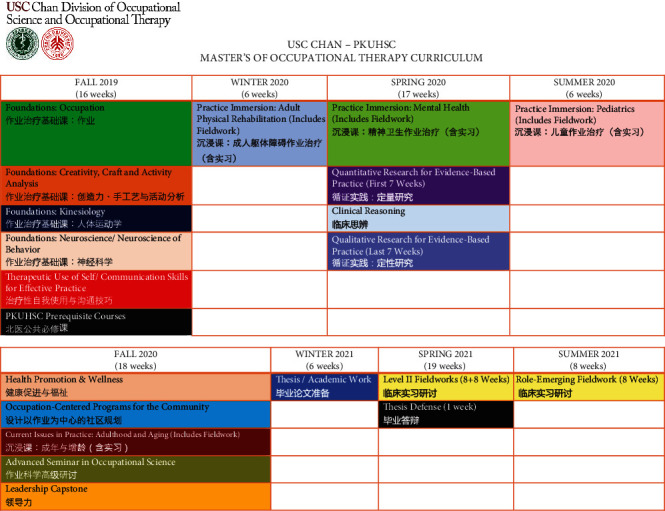
Scope and sequence (“blueprint”) of the two-year master's program in rehabilitation therapy (OT track) at PKUHSC.

**Figure 3:**
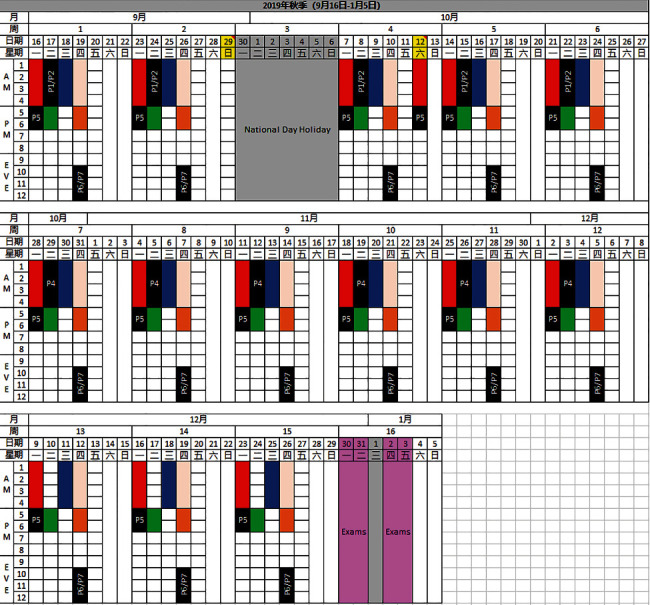
Weekly calendar spreadsheet for fall 2019. Each day consists of 12 periods of 50 minutes each, and courses are color-coded as per the blueprint above.

Meetings at PKUHSC specifically focused on an overview of their institutional academic calendar, typical dates and length of a master's program, and the PKUHSC “general” graduate school educational requirements. Half a year later, once the degree type (research master's) and the duration of the program (2 years full-time) were confirmed, faculty from both institutions together decided on the courses to include, reflecting the USC professional master's program and PKUHSC requirements. Based upon the short timeline of the collaboration and target date for launch of the program at PKUHSC, all agreed to initially retain the basic scope and sequence of the USC Chan curriculum and create cultural adaptations within each course, with the understanding that the curriculum overall would continue to be revised and culturally adapted over time throughout its delivery. An in-depth calculation of contact hours was completed in order to ensure adequate time for research work, given that PKUHSC master's students were required to complete a thesis in addition to the professional course work and fieldwork. Calculations also ensured the 24 full-time weeks of level II fieldwork required by ACOTE to plan for the students' eligibility for registration, licensure, and practice experiences in the U.S. upon matriculation into the postprofessional OTD program at USC. The curriculum also accounted for an additional 8.5-9.5 units of course work required for all PKUHSC graduate students covering topics such as common and professional English, research ethics, statistics, and politics and ideology. The outcomes of this work included the “blueprint,” or scope and sequence of courses in the PKUHSC master's program (see Figure 2), and a schedule of all occupational therapy course sections and PKUHSC prerequisite courses, mapped onto the PKUHSC Graduate School calendar, for the first cohort of master's students who would begin the program in fall of 2019 (see Figure 3).

Concurrent with the above work, two PKUHSC students were selected to come to USC for training in occupational therapy and preparation for their role as the inaugural instructors of the PKUHSC master's program. These students were selected collaboratively through a process whereby PKUHSC faculty recommended applicants; USC faculty reviewed the applications and interviewed 7 candidates, narrowing the selections to three finalists; and PKUHSC selected the final two out of the three. Because both selected students had completed training in rehabilitation therapy or rehabilitation medicine and not occupational therapy specifically, they completed the entry-master's program at USC and the postprofessional doctorate. Their doctoral work included core and elective courses along with individualized residencies curated to offer experiences in pedagogy and curriculum development, in preparation for their instructional roles. Throughout their time at USC, the two students were asked to regularly keep notes and also met regularly with members of the *China Initiative* team to discuss their reflections on the course work and implications for culturally tailoring courses for the master's courses in the PKUHSC program.

### 3.2. Conversion of the USC Entry-Master's Curriculum Model

Following decisions on the overall program length and requirements, the next step was to culturally adapt the curriculum model for the PKUHSC program in order to guide course-specific cultural adaptations. In preparation, the team reviewed several documents to inform the content essential to training OT students at PKUHSC: the mission and vision of PKUHSC; the Philosophical Base of Occupational Therapy [9]; the AOTA Philosophy of OT Education [10]; the AOTA Model Curriculum, now known as the Occupational Therapy Curriculum Design framework [11]; and the World Federation of Occupational Therapy (WFOT) and ACOTE academic program standards [3, 7].

Through a series of iterative meetings and conversations, the USC Chan *China Initiative* team and the inaugural instructors of the PKUHSC program, who at the time were current Chan graduate students, met to review the USC entry-master's curriculum model and brainstorm cultural adaptations relevant to the PKUHSC context. Using a *backward design* approach, the program outcomes and curriculum threads, or key concepts emphasized throughout all courses within the curriculum, were identified for the PKUHSC master's program prior to adapting each course. The initial program outcomes for the PKUHSC version of the curriculum included *innovator*, *leader*, *professional scholar*, and *social contributor*; the initial curriculum threads included *collaboration*, *critical thinking*, *cultural responsiveness*, *evidence-based practice*, *innovation*, *leadership*, *occupation*, and *service*. Subsequently, the USC Chan *China Initiative* team worked on reviewing literature related to these concepts to draft the description for each outcome and thread. In the final version of the PKUHSC curriculum model, three program outcomes from the USC model were retained, as they were equally relevant and important to future graduates of the PKUHSC program: *leader*, *innovator*, and *evidence-based practitioner*. The fourth program outcome, *life-long learner*, was replaced by *social contributor* to emphasize the need for graduates to provide culturally relevant services and give back to society. Five curriculum threads were retained, *occupation*, *leadership*, *innovation*, *evidence-based practice*, and *critical thinking*, and four were added, *integrity and kindness*, *collaboration*, *cultural responsiveness*, and *client-centeredness*, to reflect the high moral expectations for healthcare practitioners and the values of harmony and compassion within the Chinese cultural context.

**Figure 4:**
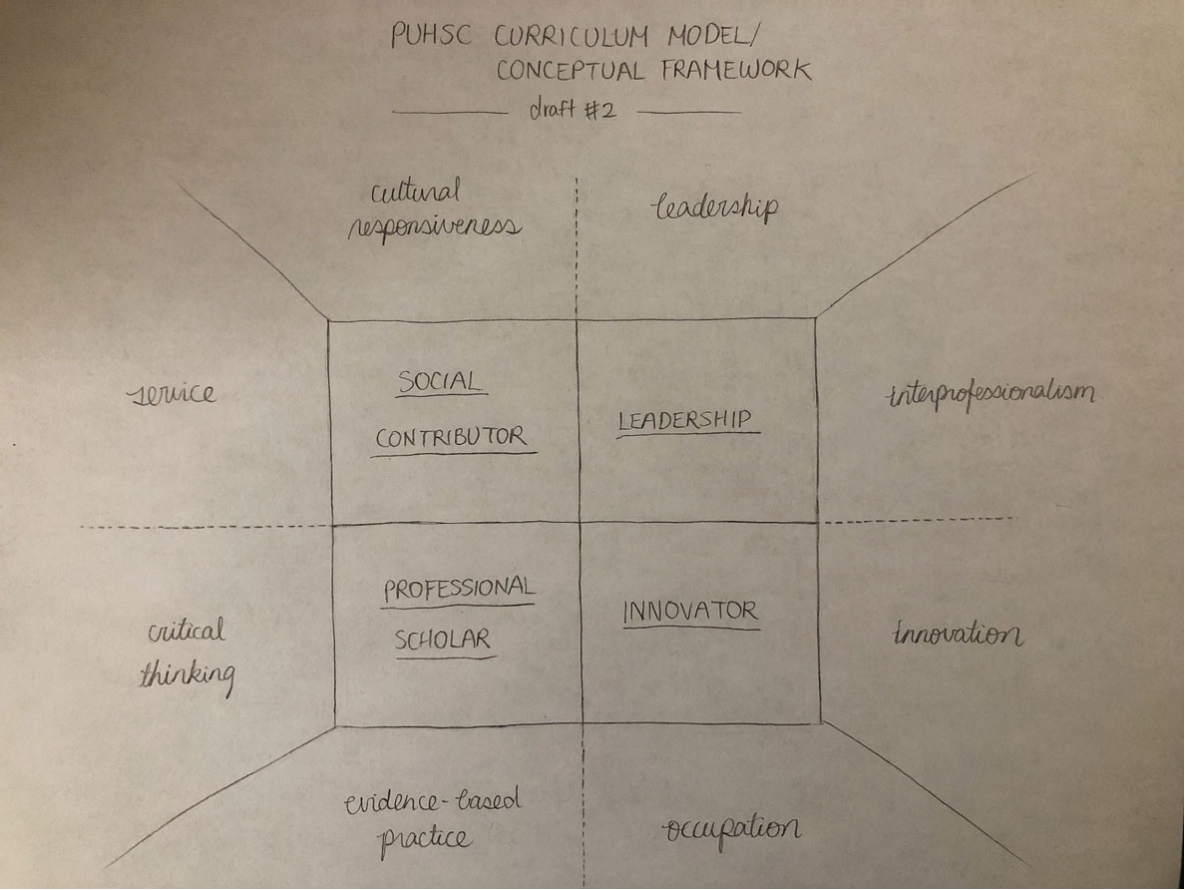
Initial draft of the PKUHSC curriculum model.

**Figure 5:**
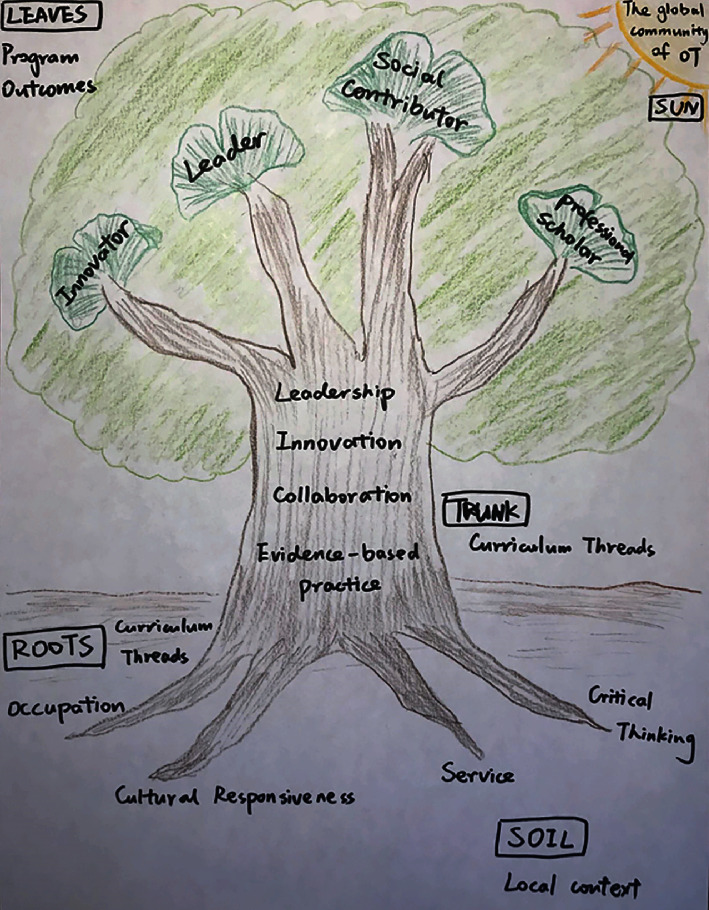
Updated schematic representation of the PKUHSC curriculum model.

Concurrent with the above process, the graphic representation of the PKUHSC program curriculum model also underwent multiple revisions. The initial draft, similar to the USC geometric model (see Figure 4), was later converted to a ginkgo tree (see Figure 5), based upon its symbolism of longevity, vitality, resilience, hope, and peace and its abundance on the Peking University campus. The ginkgo model was reviewed by faculty members from PKUHSC, together with the description of the program outcomes and threads. Ultimately, both the schematic representation of the model and the wording of some outcomes and threads were modified to be more similar to USC's concise and artistic graphic, as well as to better align with the Chinese culture and language conventions. The final results of this iterative work, along with an explanation of the schematic significance and an abridged description of each program outcome and curriculum thread, are outlined below.

#### 3.2.1. PKUHSC Master's Program: Curriculum Model

**Figure 6:**
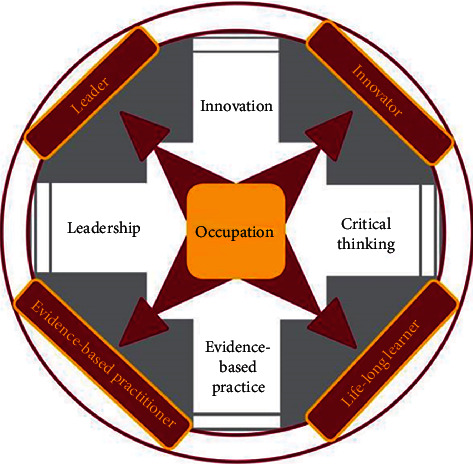
Model of the USC Chan occupational therapy program curriculum (USC Chan [15], p. 37).

**Figure 7:**
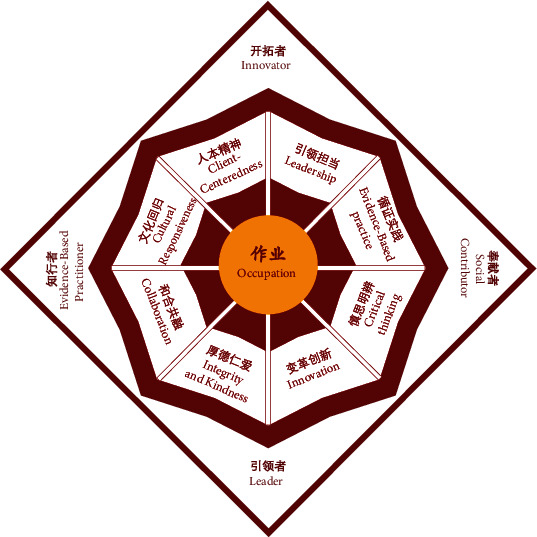
Model of the PKUHSC occupational therapy program curriculum.

The final PKUHSC master's program curriculum model is a collaborative cultural adaptation of the USC Chan entry-level master's program curriculum (see Figure 6) in order to create a graduate-level occupational therapy program at PKUHSC that is rooted in the Chinese local context and meets the occupational needs of the Chinese society. The dark red of the PKUHSC model reflects the Peking University logo, and the octangular design is inspired by the top view of the pagoda Boya, one of the landmarks of the Peking University campus (see Figure 7). In alignment with the USC model, “occupation” is at the core, represented in cardinal and gold (colors of the USC logo), further reflecting the collaboration between the two universities. The PKUHSC curriculum threads are represented as the eight beams of the pagoda: client-centeredness, leadership, integrity and kindness, innovation, cultural responsiveness, collaboration, evidence-based practice, and critical thinking. The four corners of the pagoda represent the overall program outcomes of the PKUHSC master's degree program, which aims at developing occupational therapy graduates who are innovators, leaders, evidence-based practitioners, and social contributors.

#### 3.2.2. Program Outcomes

Graduates from the PKUHSC Master's degree program in Occupational Therapy will be as follows:

*Innovators*. Graduates are *creative problem-solvers*, who implement *occupation-centered practice* in current and emerging areas

*Leaders*. Graduates are articulate, well-spoken *advocates* who convey the unique value of occupational therapy, occupational science, and occupation-centered practice. Graduates are leaders and *collaborators* on interdisciplinary teams

*Evidence-Based Practitioners*. Graduates use *evidence* to support their practice and are *self-reflective practitioners* who are empowered and committed to their own learning and professional development

*Social Contributors*. Graduates are socially responsible citizens who provide service to *meet the occupational needs of society* and are *culturally responsive* to the unique cultural makeup of Chinese society

#### 3.2.3. Curriculum Threads (Descriptions Abbreviated from the USC-PKUHSC Student Handbook (2022))

*(1) Client-Centeredness*. As a profession that sees human beings as conscious, active agents [12], the elements of client-centered practice have been present in occupational therapy since the inception of the profession [13]. Clients are the experts of themselves, their own lives, and occupations. Therefore, occupational therapists, in their practice and research, consider clients as the center of the therapeutic process and as active participants rather than the passive recipients of services.

*(2) Leadership*. Leadership within the field of occupational therapy is evidenced by knowledge of occupational science, contribution to the expansion of the profession in current and emergent practice settings, and skills in transformational leadership. As described by Northouse [14], this type of leadership takes into consideration the desires and needs of others and aims at promoting others to achieve higher levels of motivation and morality (p. 20, 186). Students learn to apply these skills with clients, colleagues, and to the profession as a whole ([15], p. 40).

*(3) Integrity and Kindness*. In the views of traditional Chinese culture, healthcare service is not only the practice of clinical expertise but also the embodiment of the spirits of integrity and kindness, which require the high moral standard of the practitioner [16]. For occupational therapists, these strong moral principles are expressed through their obligation to help people experiencing occupational injustice, as well as their dedication to the community and society overall. Occupational therapists have a role and responsibility to address barriers and injustices related to participation in occupation for all individuals and communities [7]. Specifically, students contribute to society by designing and implementing community-based projects in areas with limited resources as well as becoming involved in the process of training future occupational therapists through fieldwork supervision or mentorship programs.

*(4) Innovation*. Anthony [17] defines innovation as “something different that has impact” (p. 18), a combination of creative thinking and doing through informed risk-taking. According to Anthony, the potential for innovation lies within every individual. In a world that is becoming increasingly dynamic, innovation is required to ensure sustainability of the occupational therapy profession. Students learn to know and express their capacities through multiple opportunities to create and take risks in the learner-centered curriculum.

*(5) Cultural Responsiveness*. As occupation is a complex, dynamic, and culture-bound phenomenon, it is essential that occupational therapy practitioners are capable of providing care that is sensitive to a person's beliefs, priorities, and preferences for managing health, illness, and disability. Muñoz [18] conceptualized culturally responsive caring in occupational therapy as five interdependent key components which include generating cultural knowledge, building cultural awareness, applying cultural skills, engaging diverse others, and exploring multiculturalism. The curriculum includes opportunities for students to learn about diverse cultural and ethnic groups in China, including their health-related beliefs, practices, and cultural values [19]. The relationship between the traditional Chinese health heritage, occupational therapy, and occupational science, as well as its implications for clinical practice and research, will be explored in coursework and classroom activities.

*(6) Collaboration*. Occupational therapists are encouraged to collaborate with clients, the community, and other healthcare professionals, as collaboration in healthcare is proven to promote quality and safety in patient care, improve participation, help to achieve intervention goals, and increase innovation [20]. The development of collaborative skills is woven throughout the curriculum. Students have the opportunity to understand the role and responsibilities of occupational therapists in an interdisciplinary team through activities including team discussion, teamwork, and interprofessional education. Interprofessional education and training is crucial for healthcare professionals in that students can increase their knowledge about other healthcare providers, enhance and carryover student teamwork abilities, address stereotypes of other professions, and increase successful collaboration in clinical practice [21].

*(7) Evidence-Based Practice*. Contemporary clinical reasoning in occupational therapy and other healthcare disciplines is informed by evidence-based decision-making [22]. Evidence-based practice reflects a process that integrates the best available research findings, clinicians' experience and expertise, and clients and families' values, preferences, and circumstances [23, 24]. Students participate in research synthesis and appraisal, self-directed learning, critical thinking, and problem-solving ([15], p. 38).

*(8) Critical Thinking*. Critical thinking encompasses a broad set of skills, including professional conduct and ethical decision-making ([15], p. 39). Critical thinking involves examining one's own assumptions in light of others' perspectives and is therefore closely linked to self-reflection and empathy [25]. Students learn to develop an independent thought process through understanding and respecting the perspective of others, particularly the perspectives of people with disabilities and those from underserved populations.

### 3.3. Cultural Adaptation of the Curriculum

#### 3.3.1. Courses and Content

Once the adapted curriculum model was drafted for the PKUHSC program, the USC *China Initiative* team of faculty and doctoral residents, along with the future PKUHSC master's program instructors, shifted their focus to course content. While the curriculum scope and sequence from USC were retained for the first version of the PKUHSC master's program, it was critical that the PKUHSC courses did not merely copy the USC Chan courses, but rather were culturally adapted for the Chinese students and context, as well as the clients they would serve.

**Figure 8:**
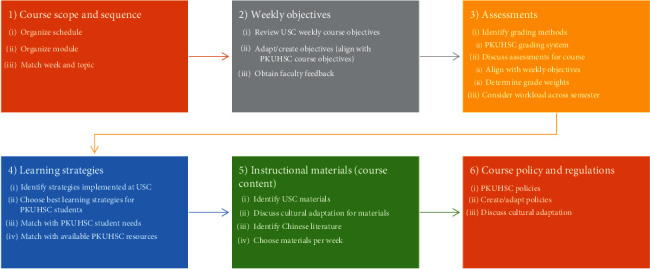
Syllabus adaptation process map.

Adaptation of course content began with review of the USC course syllabi, with particular attention to the cultural relevance of the course titles, descriptions, and overall learning objectives. Based upon team analysis and consensus, the course titles, descriptions, and course-level learning objectives were revised to better fit the Chinese cultural context. This process involved extensive dialogue and literature review to determine which content was core to the course, which topics needed cultural adaptation, and what additional objectives were needed. After reaching initial consensus on cultural adaptation of the course descriptions and overall learning objectives, each course syllabus was further modified according to the detailed steps outlined in Figure 8.

USC Chan instructors who taught the foundational courses were consulted and provided input on which course content was crucial, the sequence of course topics, and any other proposed edits. Course readings were also adapted; key OT literature was retained, while others were replaced with readings more relevant to the Chinese population. The level of difficulty of the content and the local availability of the readings were also considered in selecting course readings for the PKUHSC program. Although pedagogical approaches are different in China, consisting more of didactic teaching, the working group decided to retain the learner-centered methods of the USC program as supported by best practices, in order to prepare these graduates for their education as dual-degree students at USC.

Along with the extensive work on cultural adaptation of the individual courses, the team prepared several documents required by the PKUHSC Graduate School for new academic programs, including course application documents; a detailed program calendar; a material inventory; a space proposal for classrooms, labs, and offices; a student handbook; and a training plan for the program. Course application documents included an academic year planning table, as well as the following 3 documents for each of the 17 courses in the program: an abridged course syllabus, a course application form, and a course guide. The material inventory and resource proposal cataloged the assessments, supplies, textbooks, and equipment needed per course. For the student handbook, the future inaugural instructors drafted the mission, vision, and philosophy for the master's program at PKUHSC. Finally, the training plan outlined the curriculum requirements, including the number of required cases for the medical conditions seen in fieldwork. By the end of August 2019, these documents, including the adapted syllabi for the foundational courses, were completed and sent to PKUHSC for review.

#### 3.3.2. Fieldwork Placements and Supervision

In addition to culturally adapting courses, the team worked collaboratively on innovative ways to ensure that the students' level II fieldwork experiences met accreditation standards. Although the master's program at PKUHSC was not accredited, either by WFOT or ACOTE, the team agreed on using the ACOTE standards as a reference for the overall educational quality of the program and also to ensure the students had the training experiences to prepare them for matriculation into the postprofessional doctoral program. Additionally, PKUHSC required the students to complete all level II fieldwork experiences at PKUHSC-affiliated sites. The sites available were predominantly hospitals, given that therapy is not typically offered in community-based settings, and also hospitals in which rehabilitation therapy was more prevalent than occupational therapy, specifically. Given the limited scope of the OT profession in China to primarily adult rehabilitation hospital settings, as well as the limited number of trained occupational therapists, the ACOTE standards most difficult to meet were C.1.10 and C.1.11.

C.1.10 Length of Level II Fieldwork states “the student can complete Level II fieldwork in a minimum of one setting if it is reflective of more than one practice area, or in a maximum of four different settings” (p. 42). The PKUHSC master's students completed three eight-week fieldwork experiences. The team was able to identify three adult rehabilitation hospitals and one pediatric hospital at PKUHSC that could accept students and, therefore, all students in the cohort rotated through these sites in order to ensure each had experiences in both adult and pediatric rehabilitation. C.1.11 Qualified Level II Fieldwork Supervisors requires fieldwork educators (FWEs) to have adequate academic preparation and at least one year of practice experience as an occupational therapist. To ensure adequate supervision, the team worked to identify FWEs who (a) graduated from a WFOT-approved program, (b) were providing occupational therapy services, vs. rehabilitation therapy, for at least some portion of their caseload, and (c) had a minimum of one-year experience as a therapist. By reviewing resumes of practitioners in the above hospitals, the team identified five therapists who met these qualifications, all of whom worked in adult rehabilitation. At the pediatric hospital, where there were no qualified FWEs on site, the minimum eight hours per week of “remote supervision” was provided by a team of practitioners with the minimum three-year practice experience, including the PKUHSC course instructors trained at USC as well as a member of the Chan Division faculty.

### 3.4. Program Support and Ongoing Evaluation

#### 3.4.1. Mentoring of Inaugural Instructors

As an integral part of this cross-institutional collaboration, faculty at USC Chan also served as mentors for the inaugural instructors in the PKUHSC master's program. This guidance and support by individuals with experience in academia and in the practice of occupational therapy was crucial to the program's success, given the nascence of the occupational therapy profession and expertise in the PKUHSC context. While there was a well-established expertise in rehabilitation medicine at PKUHSC and extensive enthusiasm and support for the conceptual foundations of occupational therapy and occupational science, the establishment of occupational therapy as a distinct discipline began with this collaboration.

The majority of the mentoring was provided by the Chan *China Initiative* team, led by the Team Director and Associate Chair for Curriculum and Faculty and including occupational therapy doctoral students from USC. The process began when the inaugural instructors matriculated into the USC entry-master's program in 2017. Throughout their participation in course work at USC, the two students were invited to participate in monthly reflective dialogues with members of the *China Initiative* team. Although both students were new to the profession of OT, one had worked for ten years as a rehabilitation therapist with a physical therapy focus at the PKUHSC Third Hospital and the other was part way through training to become a rehabilitation physician. They were specifically asked to keep reflective notes on the concepts they were learning in the master's program, especially with respect to their applicability or inapplicability to the Chinese rehabilitation and cultural context. Their observations were the content of these regular meetings and also gave rise to initial ideas for cultural adaptation of the curriculum content.

In addition to these reflective meetings throughout their time in the master's program, as each student moved forward into the postprofessional OTD program, the team designed curated OTD residency experiences for each student to best prepare them for their future role as occupational therapy instructors. Rather than engage in a residency track focused on one practice area as was common for USC OTD residents, these students engaged in residencies that combined activities related to pedagogy, curriculum design, and clinical practice to bolster their understanding of best practices in teaching, course development, and instructional design. They were simultaneously engaged with the *China Initiative* team on applying finishing touches to syllabi to be used in the first semester of the PKUHSC master's program, a process that included additional mentoring by select Chan faculty experts related to course content.

At the launch of the program, the mentoring team traveled to Beijing and was joined by leading faculty members from PKUHSC in providing additional guidance and feedback during a live teaching demonstration at PKUHSC. Subsequent to the program launch in 2019, the instructors began teaching the fall semester courses and the PKUHSC instructors and the USC Chan *China Initiative* team met weekly over Zoom to dialogue regarding many topics related to serving as novice instructors, some of which included course design, classroom management, assessment, professional development, faculty roles, and loads, to name a few. Weekly Zoom meetings continued throughout the entire two years of the first cohort of PKUHSC master's program and then decreased in frequency to one or two times monthly over the subsequent years. These meetings became even more crucial as these new instructors migrated the entire new curriculum to an online teaching platform in response to and through the COVID-19 pandemic. The teams continue to meet monthly, and the focus has gradually shifted from mentoring to program evaluation and revision along with future planning.

#### 3.4.2. Implementation of an Annual Curriculum Review Process and Data Gathered

As part of the ongoing mentoring process, the PKUHSC instructors and the Chan *China Initiative* team collaboratively developed a plan for systematic evaluation of the program, through design and implementation of an annual curriculum review and systematic collection of data to support curriculum revisions. The team reviewed the form typically used at PKUHSC to gather student input at the end of each course and also created and distributed a program evaluation survey to all students in the first cohort, based upon the form used annually by the Chan Division to gather student feedback on their academic preparation for fieldwork. The first annual review was held virtually in August 2021 with members of both teams and included summary presentations by the PKUHSC instructors on the course and instructor evaluations from year one, as well as the program evaluation survey. Curriculum changes resulting from this initial year-one review are discussed below and addressed both the overall scope and sequence of the curriculum and specific course-related changes, such as content and assessment methods. The PKUHSC instructors also developed a plan for integrating ongoing program evaluation efforts into their activities and annual calendar.

From June to October of 2022, the PKUHSC instructors then conducted an independent review of the curriculum which included a survey and interviews. They sent a program evaluation questionnaire to all 19 students across the three cohorts and interviewed four instructors and faculty mentors who participated in and implemented the program. The questionnaire covered seven areas including (1) program objectives and purpose; (2) clinical education; (3) teaching and assessment methods; (4) curriculum and content; (5) recruitment, employment, and policies; (6) faculty load and qualifications; and (7) teaching resources and support. Eighteen (18) students responded and results were overall positive. Recommendations to enhance the program included continued cultural adaptation, strengthening of research content and experiences, expanded diversity within fieldwork education, integration of local clinicians with practice expertise, further professional development of the instructors, enhanced educational facilities and resources, and increased outreach and recruitment to promote the program overall [26].

#### 3.4.3. Revisions to Date

Since the initial curriculum proposal with the program launch in 2019, PKUHSC has undergone several rounds of curriculum revision stemming from the review processes outlined above. Some of the changes were at the programmatic level, and others involved changes within specific courses. Below is a nonexhaustive list of program changes that were made or are being proposed at the time of writing of this article. The majority of the programmatic changes reflect efforts of the two teams to settle on a curriculum design that meets standards of graduate professional education along with the master's degree level requirements in China, which include a thesis project to be completed by all students.

Changes already implemented focused on scheduling updates to accommodate the students' thesis project requirements along with further cultural adaptations of course learning materials and experiences. Level II fieldwork experiences were moved earlier in the curriculum to accommodate thesis defense and graduation deadlines, and a week entirely without classes was scheduled in the middle of the program to allow instructors to focus on curriculum review and students to work on their theses. Pedagogical and cultural adaptations included administering knowledge quizzes after class sessions rather than at the start, per student request; using translated versions of key textbooks and textbooks published in Chinese; adding lectures on influences between Chinese culture (Daoism, Buddhism, Confucianism, etc.) and occupation, as well as reimbursement and health policies from China; and including guest lectures and case examples from local therapists.

One example of a cultural adaptation stemmed from scholarly work led by one of the inaugural instructors who graduated from the USC master's and OTD programs. To better reflect occupation in the Chinese cultural context, Liu et al. developed the model of occupational harmony [27, 28], based on traditional Chinese culture and Chinese scholars' human complex system theory [29], a contrast to the occupational science concept of “occupational balance” [30, 31]. The content was added to two courses, *Advanced Occupational Science Seminar* and *Health Promotion and Wellness*, wherein OT students at PKUHSC learned about traditional Chinese beliefs on occupation and health and applied the model to practice in their local contexts. Additionally, joint classes were held for students at PKUHSC and USC Chan students to develop global and cross-cultural understandings of occupational harmony and occupational balance. These revisions received positive feedback from both students and instructors, as they helped the students understand how the orchestration of everyday occupations relates to health and well-being in a way that is meaningful to them and lays the foundation to inform more culturally responsive occupational therapy practice.

#### 3.4.4. Future Directions

In the future, the length of the PKUHSC master's program will increase from two years to three years in order to accommodate all course work and fieldwork and allow ample time for students to complete their thesis project. Specifically, the practice immersion courses and corresponding level I fieldwork, as well as the level II fieldwork, will be spread across six semesters to reduce workloads and allow breaks for both students and instructors. Additionally, the research courses will be moved to the first semester to better equip students with the knowledge and skills necessary for the development of their research proposals, which are due early in the program. Other planned programmatic modifications will include shifting course work to better reflect the essential knowledge and skills required for competent occupational therapy practice in the Chinese context, such as combining the *Leadership Capstone*, *Clinical Reasoning*, and *Communication Skills* courses into one course named *Occupational Therapy Professional Competencies*, merging content of *Occupation-Centered Programs for the Community* and *Health Promotion and Wellness*, and adding a seminar on *Rehabilitation Research.*

On a final note, WFOT approval for the PKUHSC master's program has been a consideration since the beginning of the partnership and continues to be a priority. The *China Initiative* team familiarized the inaugural instructors with the WFOT application process and approval standards during their studies at USC, and one of the instructors began writing elements of the WFOT application as part of their doctoral residency portfolio. In preparation, the core teams from both institutions also worked to ensure that all PKUHSC program components adhered to the quality standards outlined by ACOTE, as a precursor to the more internationally relevant WFOT standards. In 2019, the two teams also met with leaders from the China Occupational Therapy Association and other schools who have received WFOT approval to gather recommendations on the application process and input on characteristics of the PKUHSC program with respect to the WFOT standards. Recommendations from the group included hiring of additional instructors with extended practice experience, continuing to ensure that fieldwork sites meet requirements, and securing sponsors to improve materials and resources allocated to this program.

## 4. Discussion and Implications for “Practice”

The development of the USC-PKUHSC dual-degree program and cultural adaptation of the master's program curriculum reflects work that spanned multiple years and was grounded in best practices in curriculum development and cross-cultural partnerships. Despite both teams' best efforts to produce a high-quality graduate program, limitations in time, material resources, and personnel made it difficult at times to deliver an ideal curriculum with a comprehensive *backward design* and cultural adaptation. While the development teams were unable to collaboratively culturally adapt the content of each course syllabus, the overarching program outcomes and curriculum threads have been collaboratively selected to guide this work-in-progress. It is important to acknowledge that the amelioration of this program is ongoing, reflecting a commitment to continuous quality improvement.

The USC and PKUHSC teams also elected not to follow all recommendations for cultural adaptations identified in the literature. In terms of pedagogy, the group intentionally decided to retain a Western approach in the delivery of the PKUHSC master's courses, as members from both institutions believed in the value of an active learning approach in fostering driven and confident future leaders. In other cross-border educational partnerships, a similar approach has proven effective and beneficial to students' learning and growth. In an exportation of medical curricula from the U.S. to Malaysia, which represents a collectivistic, Confucian-based learning environment, students noted adapting to a more active and interactive classroom, a curriculum more focused on knowledge integration over rote memorization, and professors who were more open and approachable [32]. Although the transition was challenging at first, students were able to adapt to the new structure and approach and voiced preference for and willingness to adapt to a “culturally dissonant style” (p. 8). Similarly, in a study of transnational higher education, Chinese students also preferred western styles of teaching such as interactive lectures and saw the traditional Chinese approach as passive and less engaging [33].

Grounded in the experiences of this multiyear collaboration between USC and PKUHSC, below is a list of recommendations to promote successful cross-cultural curriculum work:

- Set clear expectations from the beginning, including the scope of the work and intended outcomes, and clear parameters for mutual responsibilities and quality improvement over time [34]. Expectations should include a comprehensive list of resources required to meet minimum standards required by approval/accreditation entities
- Address logistical factors with the same level of attention as the quality of the curriculum itself. Contextual factors may hinder or facilitate this collaboration, such as the academic calendar of the institution (e.g., length of semesters and major holidays), institutional academic requirements (e.g., general education courses), teaching and office spaces, and course materials and supplies
- Arrange multiple in-person visits, in both settings, in order to get to know one another and to understand each other's culture and context. Cultural adaptation is not simply a language translation but, more importantly, the contextualization to local cultures [35]. Frequent exchanges breed familiarity and comfort to share ideas, brainstorm through problems, and question existing structures through a balance in both knowledge-sharing and teaching and learning practices to achieve “cultural synergy” ([36], p. 633)
- Employ a *backward design* framework and principles to structure the process, as these guidelines can facilitate cultural adaptation via a focus on learning outcomes as a beacon
- Intentionally involve the personnel “on the ground” in the development process (those responsible for implementing/delivering the curriculum), as they are the most familiar with the local context and can serve as a bridge between the initial plan and any necessary further modifications, based on student input and their experience
- Develop a reliable and ongoing mentoring relationship. Continuous mentoring is paramount, and a supportive learning environment speaks not only to student-centered learning but also physical resources and infrastructure to support teacher development [33]
- Maintain flexibility in order to work with different policies and regulations, such as institutional parameters with respect to fieldwork placements
- Include a framework for ongoing program evaluation and revision from the beginning
